# Plant Sterol-Enriched Palm Oil Intervention to Improve Lipid Profile and Inflammation Status in Hyperlipidemic Individuals

**DOI:** 10.3390/nu16193370

**Published:** 2024-10-03

**Authors:** Mira Dewi, Drajat Martianto, Nuri Andarwulan, Renata Kazimierczak, Dominika Średnicka-Tober

**Affiliations:** 1 Faculty of Medicine, IPB Darmaga Campus, IPB University, Bogor 16680, West Java, Indonesia; mirade@apps.ipb.ac.id; 2 Southeast Asian Food and Agricultural Science and Technology (SEAFAST) Center, IPB Darmaga Campus, IPB University, Bogor 16680, West Java, Indonesia; dmartianto@apps.ipb.ac.id; 3 Department of Community Nutrition, Faculty of Human Ecology, IPB Darmaga Campus, IPB University, Bogor 16680, West Java, Indonesia; 4 Department of Food Science and Technology, Faculty of Agricultural Technology, IPB Darmaga Campus, IPB University, Bogor 16680, West Java, Indonesia; 5 Department of Functional and Organic Food, Institute of Human Nutrition Sciences, Warsaw University of Life Sciences, Nowoursynowska 159c, 02-776 Warsaw, Poland; renata_kazimierczak@sggw.edu.pl

**Keywords:** plant sterols, phytosterols, palm oil, cholesterol, LDL, C-reactive protein, hypercholesterolemia, Indonesia

## Abstract

**Background:** Cardiovascular diseases, including coronary heart disease (CHD), are currently positioned among the leading causes of mortality globally. Risk factors of CHD include, among others, hypercholesterolemia and elevations in systemic inflammation. Functional foods enriched with compounds showing cholesterol-lowering effects are considered one among various dietary and lifestyle intervention strategies to tackle this problem. A CHD-preventive effect of dietary plant sterols has been broadly discussed, not only due to their ability to reduce blood cholesterol level, but also to their proven anti-inflammatory potential. Palm oil is one of the most widely consumed edible oils in the world. Despite its widespread use, especially in Asian countries, no study has been conducted using palm oil as a vehicle for plant sterols. **Methods:** The aim of the placebo-controlled double-blinded trial presented here was, therefore, to evaluate the effect of palm oil enriched with plant sterols, used as a cooking oil, on lipid profile and systemic inflammation marker in 100 adult hyperlipidemic residents of Bogor, Indonesia. **Results:** The study has shown a significant reduction in total cholesterol and LDL cholesterol level in study subjects consuming plant sterol-enriched palm oil as a replacement for usual palm oil for cooking, with no similar effect on CRP levels. **Conclusions:** The study suggests that, along with a healthy diet and lifestyle promotion, incorporating plant sterols in palm oil used for cooking may be an effective strategy to reduce cardiovascular risks in hyperlipidemic individuals.

## 1. Introduction

Cardiovascular diseases (CVDs) are currently considered among the leading causes of mortality globally, with their prevalence rising dramatically, especially in middle- and low-income countries and territories [1,2,3,4]. In Indonesia, CVDs, including coronary heart disease (CHD), are estimated to cause over 30% of deaths [2,5], positioning the country in fourth place worldwide for the highest rates of CVD-associated mortality [2,4], as well as having a high number of disability-adjusted life years lost to CHD [6,7].

The risk factors of CHD include, among others, lipid aberrations (i.e., elevated blood cholesterol), hypertension, abdominal adiposity, diabetes, and elevations in systemic inflammation [3,8,9]—most of them to a great extent associated with an unhealthy diet, lifestyle, and stress [3,10].

It has been evidenced that LDL cholesterol is one of the significant factors contributing to the development of atherosclerosis, and thus leading to CHD [11,12,13]. Therefore, reduction of blood LDL cholesterol levels is one of the target strategies to reduce the risk of CHD and other diseases associated with hypercholesterolemia [14,15]. In Indonesia, cholesterol level abnormalities have been detected in over 30% of the adult population [5].

Along with the increasing incidence of CHD among productive age groups, many studies have been carried out on its prevention and treatment using food-based as well as pharmacological approaches. Clinical trials, predominantly in hypercholesterolemic subjects, have indicated the cholesterol-lowering effect of dietary supplementation with plant sterols [16,17]. Plant sterols and stanols, also known as phytosterols, have been found to be effective in reducing the absorption of both dietary and biliary cholesterol from the intestinal tract. Several specific mechanisms of their action in lowering blood LDL cholesterol have been scientifically proven [18,19]. Besides their ability to reduce blood cholesterol level, recent studies have shown that the cardioprotective effect of dietary plant sterols is mediated by their ability to reduce inflammation that underlies the disease [20,21]. It has been estimated that a clinically significant effect of plant sterols can be achieved with an intake of 1.5 to 3 g a day [18,22].

Although several food products enriched with plant sterols are available on the market, more affordable, safe, and effective plant sterol vehicles for hypercholesterolemia prevention are still to be found [23]. Most clinical studies focusing on plant sterol interventions have been conducted using plant sterol-enriched spreads, yogurt, margarine, beverages, fish oils, bread, low-fat milk, etc. [24,25]. To the best of our knowledge, no study has been conducted using palm oil as a vehicle for plant sterols.

Palm oil, obtained from the fruit of the oil palm tree (*E. guineensis*), is one of the most popular edible oils globally, produced mainly in Indonesia and Malaysia, but used by virtually all households and manufacturing industries worldwide [26]. Next to coconut oil, palm oil is one of the few highly saturated vegetable oils, semisolid at room temperature. It is a common cooking ingredient in the tropical parts of Africa, Southeast Asia, and some regions in Brazil. Its use in the food industry in other regions of the world is also widespread, mostly due to its low cost [21], but also the high oxidative stability of the refined product during frying [27]. In 2022–2023, global production of palm oil was estimated to have reached 78 million metric tons and is projected to grow to up to 240 million metric tons by 2050 [28]. Indonesia is the world’s largest producer of palm oil [26,29]. It has been reported that 100% of Indonesian households consume palm oil as cooking oil, and the average consumption of palm oil as cooking oil reached 23 g/day [30,31].

As fats are needed to solubilize sterols, palm oil seems to be an ideal vehicle to increase lipid solubility and facilitate the incorporation of plant sterols into micelles.

Based on the considerations mentioned above, the main objective of the presented study was to evaluate the effect of palm oil enriched with plant sterols, used as cooking oil, on lipid profile and systemic inflammation marker in hyperlipidemic individuals. The study included the analysis of the nutrient intake and nutritional status of subjects, the analysis of the effect of the palm oil intervention on blood lipid profile (total cholesterol, LDL and HDL cholesterol, and triglycerides), and on systemic inflammation as assessed by blood c-reactive protein (CRP) level.

## 2. Materials and Methods

### 2.1. Study Design

The study was designed as a double-blinded randomized controlled trial, with an eight-week intervention period, and conducted in Bogor District, West Java, Indonesia. The study subjects were volunteers residing in Bogor City and Bogor District, with known mild–moderate hypercholesterolemia, who responded to the study invitation and who fulfilled the following inclusion criteria for the study: men and women aged 25–60, total plasma cholesterol concentration at screening ≥200 mg/dL, not requiring lipid-lowering drug therapy during the trial. The exclusion criteria included secondary hyperlipidemia, fasting triglyceride concentration >3.5 mmol/L, body mass index >35 kg/m^2^, use of any lipid-lowering drug, suffering from gastrointestinal diseases or severe concomitant diseases, and unwillingness to participate.

The trial was designed to have 80% power with a confidence of 95% to detect a difference of 6% in total cholesterol concentration between the intervention and placebo groups at the end of each treatment period for each of the two groups of subjects. The sample size was calculated using the following formula:

$$n=2\sigma^{2}[Z_{1-\alpha /2}+Z_{1-\beta }{]}^{2}/{\left(\partial \right)}^{2}$$

where:

n—sample size per group
$\sigma^{2}$—variance of either group (assumed to be 11% and equal for both groups)
∂—minimal detectable difference between the two means (6%)
$Z_{1-\alpha /2}\;and\;Z_{1-\beta }$—standard normal deviates at an α level of significance and at a 1−β power, respectively. $Z_{1-\alpha /2}$ is 1.96 at a 5% level of significance for two-sided tests, while $Z_{1-\beta }$ is 0.84 at 80% power.

To consider a potential dropout rate of 15%, 50 subjects per group (100 in total) were recruited. The recruitment procedure included the following steps: those who met the age criterion and were found to have a total plasma cholesterol level of ≥200 mg/dL (screened by fingertip test using a standard cholesterol home test kit—CardioChek PA analyzer, PTS Diagnostics ©, Indianapolis, IN, USA) were invited to participate. The purpose of the trial was explained, and informed consent was obtained from all participants. Eligible participants were randomly assigned to the treatment and control groups. The researchers and subjects remained blinded to individual subject assignment throughout the study. The study protocol was approved by the Ethical Committee of the Faculty of Medicine, Diponegoro University, Indonesia (protocol code No. 333/EC/FK/RSDK/2012, approval date 8 October 2012). The trial was registered in the ClinicalTrials.gov database (RCT Registration Code: NCT06595472).

### 2.2. Treatment Palm Oil

The study treatment and control cooking oil had the same fatty acid composition and were prepared following the same conditions and packed in individual blinded 2000 mL bottles. The enriched palm oil contained 52 mg/L of phytosterols to reach the estimated intake of plant sterols in the treatment group at around 2 g/day [22]. The phytosterols used were Vegapure 95 FF^®^ (BASF SE, Ludwigshafen, Germany) derived from soybean derivatives containing campesterol, stigmasterol, and beta-sitosterol. The treatment and control cooking oil were indistinguishable in appearance and taste. Subjects were supplied with either a control or treatment cooking oil bottle every week, to be consumed as a substitute for usually used cooking oil.

### 2.3. Data Collection and Analysis

The types of data collected in the study are listed in Table 1 below. Data on food consumption were collected every week during the intervention. Data obtained by 2-day (2 × 24 h) food recall were converted to grams, and then the daily intake of energy and macronutrients was calculated using the Indonesian Food Composition Data (DKPI) [32].

Nutritional status was determined by BMI, W/H assessment, and body composition (measured using Omron^®^ HBF 306 body fat analyzer, Omron Healthcare Co., Ltd., Kyoto, Japan), which were assessed at baseline and endline of the intervention. Blood samples for biomarker analysis were collected at weeks 0 and 8 of the intervention period after a 12-h fast. Lipid profile (triglycerides, total cholesterol, LDL cholesterol, and HDL cholesterol) was analyzed using an enzymatic colorimetric method using cholesterol oxidase para aminophenazone (CHOD-PAP). Serum high-sensitivity CRP (hsCRP) levels were analyzed using an enzyme-linked immunosorbent assay (ELISA).

For hypothesis testing, paired and unpaired *t*-tests were used. *p*-values of less than 0.05 were considered statistically significant. All statistical analyses were performed using SPSS PASW v. 18 software (SPSS Inc., Chicago, IL, USA).

## 3. Results

**Figure 1:**
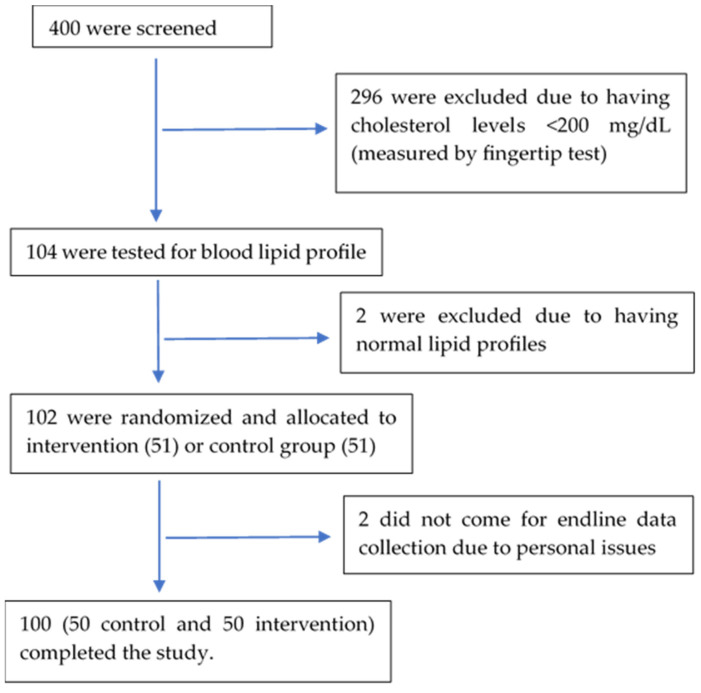
Study subject diagram.

Among the 400 residents aged 25–60 in the study area screened for hypercholesterolemia, 104 were found to have a blood cholesterol level ≥200 mg/dL by fingertip test and were analyzed further for full blood lipid profiles. Two of them were found to have normal blood lipid levels, and 102 went through randomization and intervention. Two study participants failed to come for endline data collection, while 100 completed the study (Figure 1).

### 3.1. Baseline Characteristics of Subjects

Baseline subject characteristics and baseline lipid profiles are reported in Table 2. No significant differences in baseline body mass index, body fat percentage, blood pressure, lipid profile (total cholesterol, total triacylglycerols, HDL cholesterol, and LDL cholesterol), or CRP levels were observed between the 2 groups.

### 3.2. Energy and Nutrient Intake

**Figure 2:**
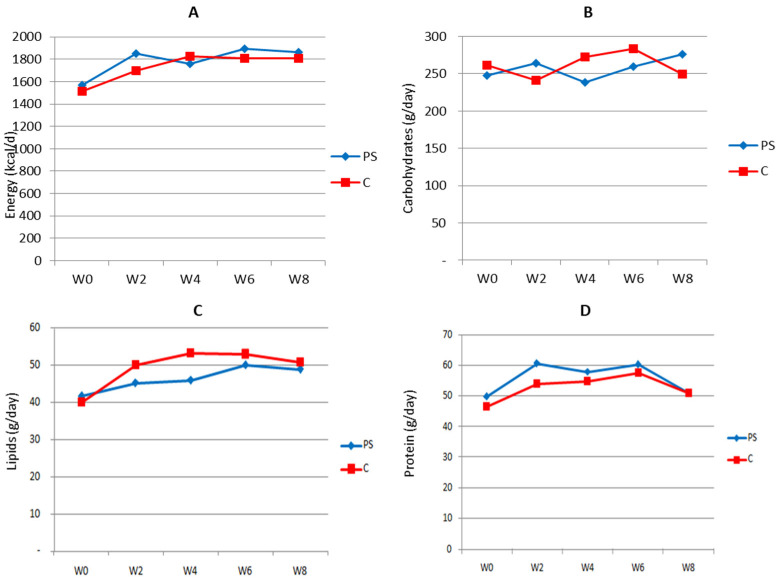
Estimated 24 h energy (**A**), carbohydrate (**B**), lipid (**C**), and protein (**D**) consumption by study subjects during the intervention period. PS = plant sterol-enriched palm oil group; C = palm oil group, control; w = week of intervention period.

The energy and nutrient intake of subjects during the study was calculated based on 2 × 24 h recall collected at baseline, as well as in weeks 2, 4, 6, and 8 of the study period. The results are presented in Figure 2 below.

Except for carbohydrates, in general, the subjects’ energy and nutrient consumption was slightly increased following the intervention but then gradually decreased to its value at baseline. As expected, the trend of consumption in both groups was similar, hence reducing its potency as bias when analyzing the effect of the intervention on biological parameters.

### 3.3. Palm Oil and Plant Sterol Consumption

**Figure 3:**
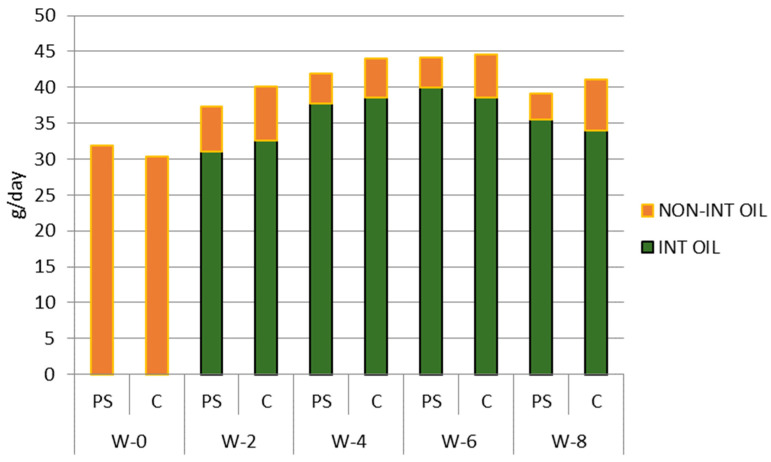
Palm oil consumption of subjects during the study period (g/day). PS = plant sterol-enriched palm oil group, C = palm oil group (control). Non int oil = cooking oil consumed from another source, not the oil given as intervention; int oil = cooking oil consumed from the oil given as intervention; w = week of the intervention period.

During the study, both groups received intervention oil, i.e., either palm oil enriched with plant sterol or palm oil (control). The compliance of subjects in consuming the intervention oil is shown in Figure 3.

The trends of palm oil consumption were similar in both groups. Compared to baseline (around 32 g/day), the palm oil consumption in both groups increased during the intervention to almost 45 g/day in week 6, and then decreased in week 8 to around 40 g/day. This similarity of palm oil consumption trends in both groups reduces their potency to introduce bias when analyzing the effect of intervention on biological parameters.

It can be seen from Figure 3 that both groups consumed comparable amounts of intervention oil. On average, about 86% of the palm oil consumed by subjects came from the palm oil given as an intervention.

**Figure 4:**
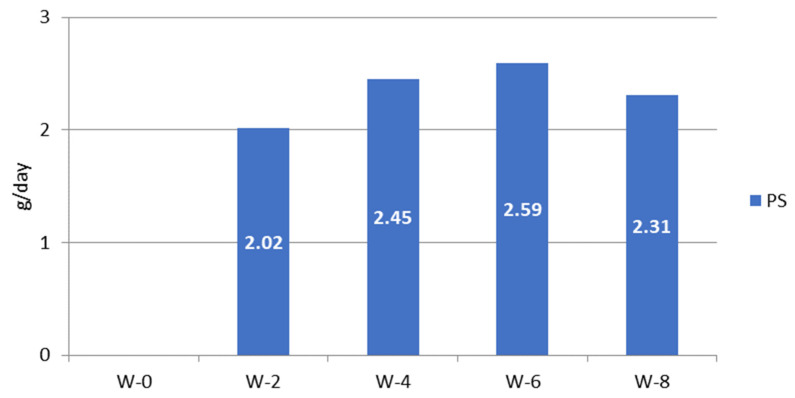
Estimation of plant sterol intake from palm oil in plant sterol-enriched palm oil group (PS) during the study; W = week of the intervention period.

The plant sterol consumption was calculated from the data on plant sterol-enriched palm oil consumed in the PS group, (Figure 4). The plant sterol intake of subjects during the study varied between 2 and 2.6 g/day, reaching on average 2.35 g/day.

### 3.4. Effect of Intervention on Lipid Profile and CRP Level

In the PS group, the blood total cholesterol level at endline was significantly lower compared to the baseline, which was associated with lower LDL cholesterol level. No other significant differences were found in the other measured blood lipid profile parameters of this group.

In the C group, no significant difference was observed between endline and baseline in blood lipids or CRP level, indicating that the palm oil given as a placebo did not have any significant effect on these parameters. Despite a statistically significant increase in the blood CRP level at the endline compared to the baseline in the PS group, the study found that there were no significant differences in the changes in blood CRP between the two groups (*p* = 0.062), as indicated in Table 3.

In the present study, plant sterol-enriched palm oil significantly reduced total cholesterol and LDL cholesterol in blood, as shown in Table 3. Compared to the C group, total cholesterol and LDL cholesterol levels were reduced by 6.08% and 7.71%, respectively.

The effects of plant sterol-enriched palm oil on blood lipid ratios are shown in Table 4. In the PS group, the total cholesterol/HDL cholesterol ratio (TC/HDL) and LDL cholesterol/HDL cholesterol (LDL/HDL) ratio at endline were significantly lower compared to baseline (*p* = 0.000 and *p* = 0.001, respectively), indicating that the intervention not only reduced the level of total cholesterol but also improved the profile of blood lipids. The differences between both groups at the endline were also significant, with *p* = 0.024 for TC/HDL and *p* = 0.028 for LDL/HDL.

## 4. Discussion

Dietary therapy is one of the key strategies to improve blood lipid profile and thereby reduce the risk of CVD. Plant sterol fortification in daily consumed foods, in addition to lifestyle modifications such as increasing physical activity, has been shown to enhance the effect of dietary therapy on lowering total cholesterol and LDL cholesterol and improving lipid profile. In the present placebo-controlled double-blinded trial, we reported significant improvement in lipid profile, as evidenced by a significant reduction in total cholesterol and LDL cholesterol level, in hyperlipidemic individuals who consumed plant sterol-enriched palm oil as a replacement for usual palm oil for cooking. To minimize individual variance in cholesterol measurements, we also analyzed blood lipid ratios, and the results confirmed the significance of blood lipid improvement in the PS group after the intervention in comparison to the control group.

The fact that plant sterols could reduce blood LDL and total cholesterol level has been shown in various studies [17,18]. Several clinical trials have established that consuming 1.3–2.0 g/day of phytosterols can result in a 10–15% reduction in LDL cholesterol in hyperlipidemic populations [18,22]. However, in the majority of studies carried out to date, the plant sterols were consumed in fat matrices such as margarine, butter, or dressing; reduced-fat matrices such as yogurt, low-fat milk, reduced-fat spread, etc., as well as non-fat matrices such as beverages; and food supplements such as capsules [33]. In this present study, we have used palm oil as a vehicle for plant sterols. Even though some recent studies have suggested that the effectiveness of plant sterols as cholesterol-reducing agents does not depend on the food matrix [34,35], fat-rich foods, such as oils or fat spreads, are considered more preferable, due to plant sterols solubilizing [36]. This fact, together with high regular daily palm oil consumption by Indonesians [31], positions palm oil among highly feasible products to be enriched with plant sterols, targeted for the Indonesian population [23]. This is additionally supported by the economic aspect of such a fortification. The most cost-effective techniques allow for the incorporation of phytosterols in food carriers such as solid and semi-solid margarine and butter, and liquid edible oils [23]. This is important to ensure that the proposed fortification intervention could benefit low-income consumers. The main significant result of the present study is that plant sterol-enriched palm oil, used as cooking oil—like usually practiced in Asian countries, especially Indonesia—is as effective as other plant sterol-enriched or fortified foods in reducing blood total cholesterol and LDL cholesterol.

Previous studies looking into the impact of palm oil on CVD risk focused mainly on palmitic and stearic acids’ effects and reported various results [37]. Some studies reported that palm oil consumption negatively affects blood lipid profile in hypercholesterolemic subjects [38]. Although not always confirmed [39], most studies support the concept that the palmitic acid in palm oil raises LDL cholesterol level. The present study showed that in the control group who received palm oil, there were no significant changes in blood lipid parameters observed after 8 weeks of intervention, even though the overall consumption of palm oil in this period increased. Different findings might be due to ethnicity and geographic differences among various populations studied. Other factors such as physical activity and diet composition also play important role in making up one’s blood lipid profile.

Increasing evidence indicates a pivotal role of inflammation in the development of atherosclerosis [40,41]. C-reactive protein (CRP), named for its capacity to precipitate the somatic C-polysaccharide of Streptococcus pneumonia, is a sensitive systemic marker of inflammation and tissue damage. Numerous prospective studies have shown that high concentrations of CRP are associated with an increased risk of cardiovascular events [42]. At the same time, numerous studies have reported that plant sterol supplementation significantly lowered CRP levels [43,44]. While fewer studies have shown a non-significant change in CRP levels following plant sterol intervention, to the best of our knowledge, no study has reported a significant increase as in the present study, in which plant sterol-enriched palm oil was shown to increase blood CRP level after 8 weeks of intervention in comparison with baseline. An alleviation of CRP concentration in blood can result from various diseases and is not specific to the process involved in cardiovascular diseases [45]. Minor changes in CRP level, as seen in our study, are likely to result from a low level of underlying chronic inflammation, which may accompany various conditions (e.g., specific dietary patterns, overweight, and obesity) [46]. Other conditions that could affect the CRP level in both groups in the study were not fully controlled, which is one of the weaknesses of the study. Further analysis that includes dietary intake of specific nutrients, physical activity, and specific health conditions of every subject should be performed to look for possible factors that may have caused this result. To further analyze the effect of the plant sterol-enriched palm oil intervention on inflammation, other parameters of inflammation should also be included, such as interleukin 6, interleukin 12, and TNFα.

Studies have reported that heat treatment of plant sterol-enriched products, including vegetable oils, resulted in increased plant sterol oxidation products (POPs), varying depending on temperature, the length of the heating process, and the product matrix [47,48]. Thanh et al. 2006 [49] conducted an experiment with various types of vegetable oil containing plant sterol and reported that 100 °C heating for 1 h did not result in a significant change in plant sterol level. On the other hand, heating the oil to 200 °C resulted in 50–60% degradation of the product. Palm oil in Indonesian cuisine is usually used for stir-frying or deep-frying. The oil temperature may reach over 100 °C with deep frying, but typically the process takes less than 1 h as the oil is used on the household level. Thus, some of the plant sterol contained in palm oil may be oxidized during the cooking process, but the amount is estimated to be low.

The present study shows the beneficial effect of using plant sterol-enriched palm oil as a replacement for usual palm oil for daily usage on blood lipid profile. However, we do not support a high intake of fried foods, especially if the cooking oil is repeatedly used, because of its adverse health effects. A low-fat, high-fiber diet should always be promoted for those with hyperlipidemic status. On the other side, palm oil-fried foods have long been an important part of Indonesians’ habitual diet, and a fast and significant reduction in their consumption is not expected. Despite its arguable effects on health, palm oil is used for cooking (frying) by almost 100% of Indonesian households. Enriching palm oil with plant sterols, which have been shown to improve blood lipid profile, is therefore thought to be one of the potential strategies to reduce the adverse effects of consuming fried foods by the Indonesian population, especially for hyperlipidemic individuals.

At the same time, it should be pointed out that while some international organizations and researchers recommend the use of phytosterols as blood cholesterol-lowering agents [16,50], others indicate a need for comprehensive clinical studies, which would allow us to thoroughly estimate any potential health risks and side effects of the long-term, regular consumption of high plant sterol doses before making further decisions on such intervention [24,51,52,53]. There are studies that have linked elevated plasma concentrations of circulating plant sterols with CVD presence [24]. Some scientific literature suggests that, similarly to cholesterol, phytosterols may accumulate in the aortic valve tissue [54,55] and thus contribute to the development of atherosclerotic lesions [56], especially in subjects with the ABCG5 and/or ABCG8 gene mutations, causing an increased absorption and/or inability to remove plant sterols from the body [57,58]. This potential for plant sterols’ accumulation in cardiovascular tissue and their causal involvement in atherosclerosis development have been a matter of scientific debate over past decades [51,52,59,60,61,62]. While several studies have reported a positive association between plasma plant sterol concentrations and the risk of atherosclerotic cardiovascular disease [51,63,64,65,66,67], others did not confirm such a relation [60,68,69] or have demonstrated phytosterols’ association with reduced cardiovascular risk [70,71,72].

## 5. Conclusions

The present study suggests that plant sterol-enriched palm oil used as cooking oil shows a potential to improve blood lipid profile, as evidenced by a significant reduction in total cholesterol and LDL cholesterol level, as well as TC/HDL and LDL/HDL ratios in hyperlipidemic individuals, but it has no similar effect on CRP level. The main important result of the present study is that plant sterol-enriched palm oil, used as cooking oil, is as effective as other plant sterol-enriched or fortified foods in improving blood lipids profile. Therefore, along with a healthy diet and lifestyle promotion, incorporating plant sterols in palm oil used for cooking could be considered among potential relevant strategies to improve lipid profile, thus reducing cardiovascular risks in hyperlipidemic individuals.

Although the study brings important insight into the potential of this functional food product, well-suited for Indonesian needs and preferences/consumption patterns, further comprehensive investigation would be of importance to confirm the efficacy and safety aspects of introducing such phytosterol-enriched product into a habitual diet, before proceeding further with such an intervention. Hereby, a collaboration of researchers, governments, and the food industry is necessary to develop effective and safe strategies and tools for hypercholesterolemia and associated CHD prevention in Indonesia.

## Figures and Tables

**Table 1:** Types of data collected in the study.

Variables	Indicators	Method
Subject characteristics	Age, medical history, education, occupation, family members, marital status	Interview/questionnaire
Blood pressure	Systole, diastole	Blood pressure monitor
Nutrient intake	Consumption of energy, carbohydrates, fat, protein	2 × 24 h food recall
Nutritional status	BMI, body composition	Weight/height (W/H) measurement, bioimpedance analyzer
Blood lipid profile	HDL, LDL, triacylglycerols, cholesterol (total)	Enzymatic colorimetric method
Inflammation state	Serum hsCRP	Enzyme-linked immunosorbent assay (ELISA)

**Table 2:** Baseline characteristics of study subjects.

Variables	Intervention Group	Control Group	*p*-Value
Female, n (%)	36 (75)	40 (77)	-
Body mass index (kg/m^2^)	28.67 ± 4.80	27.10 ± 5.00	0.36
Body fat percentage (%)	35.38 ± 6.10	33.50 ± 5.70	0.53
Blood pressure—systole (mmHg)	146.77 ± 27.10	141.50 ± 27.20	0.58
Blood pressure—diastole (mmHg)	90.48 ± 14.50	87.90 ± 12.40	0.47
HDL (mg/dL)	48.02 ± 9.00	46.40 ± 9.10	0.63
LDL (mg/dL)	141.84 ± 31.50	140.60 ± 30.60	0.65
Cholesterol (total) (mg/dL)	216.07 ± 36.20	216.50 ± 31.20	0.37
Triacylglycerols (mg/dL)	152.16 ± 81.30	151.60 ± 82.60	0.79
CRP (mg/L)	2.38 ± 2.40	2.90 ± 3.40	0.55

Intervention group—receiving plant sterol-enriched palm oil; values are reported as means ± S.D; *p*-values refer to the difference between groups.

**Table 3:** Effect of plant sterol-enriched palm oil intervention on blood lipid profile and CRP level.

Parameter	Group	Baseline	Endline	*p*-Value
Cholesterol (total) (mg/dL)	PS	216.07 ± 36.20 ^a^	208.50 ± 40.66 ^b^	0.003
	C	216.50 ± 31.20	226.69 ± 36.79	
Triglycerides (mg/dL)	PS	152.16 ± 81.30	132.60 ± 69.14	0.357
	C	151.60 ± 82.60	148.27 ± 72.11	
HDL cholesterol (mg/dL)	PS	48.02 ± 9.00	49.10 ± 11.01	0.083
	C	46.40 ± 9.10	48.25 ± 9.37	
LDL cholesterol (mg/dL)	PS	141.84 ± 31.50	132.17 ± 35.49	0.027
	C	140.60 ± 30.60	144.06 ± 33.16	
CRP (mg/L)	PS	2.38 ± 2.40 ^a^	3.48 ± 3.77 ^b^	0.062
	C	2.90 ± 3.40	3.00 ± 4.71	

PS = plant sterol-enriched palm oil group; C = control palm oil group. Values are reported as means ± S.E.M.; *p*-values refer to the difference between the change of means (endline–baseline) in the PS vs. C group (tested with independent *t*-test). Means in a row with different superscript letters are significantly different, *p* < 0.05 (ANOVA with paired *t*-test).

**Table 4:** Effect of plant sterol-enriched palm oil intervention on blood lipid ratios.

	PS			C			*p*-Value **
	Baseline	Endline	*p*-Value *	Baseline	Endline	*p*-Value *	
**TC/HDL**	4.73	4.56	0.000	4.80	4.82	0.786	0.024
**LDL/HDL**	3.08	2.88	0.001	3.13	3.10	0.547	0.028

PS = plant sterol-enriched palm oil group; C = Control palm oil group; TC = total blood cholesterol. Values are reported as means; * *p*-values refer to the difference between endline vs. baseline within one group; ** *p*-values refer to the difference between PS vs. C group at endline.

## Data Availability

Data will be made available upon request by author Nuri Andarwulan.
